# CD4 T_RM_ Cells Following Infection and Immunization: Implications for More Effective Vaccine Design

**DOI:** 10.3389/fimmu.2018.01860

**Published:** 2018-08-10

**Authors:** Mieszko M. Wilk, Kingston H. G. Mills

**Affiliations:** Immune Regulation Research Group, School of Biochemistry and Immunology, Trinity Biomedical Sciences Institute, Trinity College Dublin, Dublin, Ireland

**Keywords:** memory CD4 T cell, tissue-resident memory T cell, infection, immunization, vaccine, protective immunity, Th1 cell, Th17 cell

## Abstract

The induction of immunological memory, which is mediated by memory T and B cells, is central to adaptive protective immunity to pathogens induced by previous infection and is the cornerstone of effective vaccine design. Recent studies in mice have suggested that memory T cells that accumulate in tissues, termed tissue-resident memory T (T_RM_) cells, play a crucial role in maintaining long-term protective immunity to mucosal pathogens. CD4 and CD8 T_RM_ cells can be induced following infection at mucosal sites or the skin, where they are maintained and poised to respond rapidly to reinfection with the same pathogen. T_RM_ cells can also be generated by vaccination, but their induction is influenced by a number of factors, including the type of vaccine, the adjuvant, and the route of immunization. Live attenuated vaccines appear to be more effective than killed or subunit vaccines at inducing T_RM_ cells and mucosal immunization, especially by intranasal route, is more effective than parenteral delivery. However, evidence is emerging that formulation of killed or subunit vaccines with novel adjuvants, especially those that generate Th1 and Th17 responses, can promote the induction of T_RM_ cells. While T_RM_ cells are also present at high number in mucosal tissues in humans, one of the challenge will be to develop methodologies for routine quantification of these cells in humans. Nevertheless, the identification of approaches for optimum induction of T_RM_ cells in mice should assist in the design of more effective vaccines that sustain protective immunity against a range of human pathogens.

## Introduction

The induction of immunological memory is central to antipathogen adaptive immunity induced by previous infection or vaccination. While circulating antibodies can confer protection against infection with certain pathogens, antibodies in the circulation and at mucosal sites usually wane over time and long-term protection is dependent on the induction of memory T and B cells. There is growing recognition that memory T cells that reside in tissues, called tissue-resident memory T (T_RM_) cells, play a crucial role in maintain long-term immunity, especially against pathogens that infect mucosal surfaces (1). T_RM_ cells were identified as cells that retained in the non-lymphoid organs with limited ability to recirculate. Tissue-resident lymphocytes constitutively express adhesion molecules and integrins that help them to remain in the tissue. These include CD44, a receptor for hyaluronic acid that can also bind to collagens or matrix metalloproteinases, and CD69, a transmembrane C-type lectin that is critical for regulating the T cell egress from lymphoid organs and retention in peripheral tissues (2, 3). CD103, αE integrin, is often expressed on intraepithelial and airway CD8 T_RM_ cells. As a receptor for E-cadherin, CD103 helps T_RM_ cells to adhere to the epithelium and be positioned on the first line of defense (4). However, the expression of CD103 by CD4 T_RM_ cells is more controversial. A study by Collins et al. demonstrated that CD103 can be expressed on CD4 memory T cells egressing from the skin and suggested that this marker may be modulated as CD4 T cells enter and leave the skin (5). In contrast, CD4 effector T cells that infiltrated and resided in the skin after primary infection with *Candida albicans* acquired expression of CD69 and CD103 (6). We have recently reported that infection with *Bordetella pertussis* induces CD69^+^ CD4 T_RM_ cells and a significant proportion of these cells stably express CD103 through the course of infection and after clearance of the bacteria (7). Following reinfection with *B. pertussis*, CD103 was rapidly upregulated on these cells, and this was not affected by treatment with FTY720, which inhibits lymphocyte egress from the draining lymph node and tissues (7). Retention of T_RM_ cells in tissues is facilitated by downregulation of CD62L and CCR7, “homing receptors” that allow T cells to enter secondary lymphoid organs, and sphingosine-1-phosphate receptor 1, which enables cells to egress from lymphoid tissues (8–10). The expression of other molecules like chemokine receptors on T_RM_ cells are often shaped by the specific tissue environmental cues.

Newborns and infants are in a greater risk from infections than adult humans. For example, high levels of morbidity and mortality have been reported in infants following respiratory infections with pathogens like influenza virus or *B. pertussis*, suggesting impaired protective immunity in infants compared with adults (11, 12). A possible explanation is that in pediatric tissues, the dominant population of T cells are naïve T cell emigrants from the thymus, whereas adult tissues contain predominantly memory T cells (13). Moreover, results from a mouse model of influenza infection have indicated that impaired protective immunity induced by previous infection or vaccination during infancy may reflect reduced generation of T_RM_ cells (14). Collectively, the emerging data on T_RM_ cells suggest that they play a critical role in long-term protective immunity induced by previous infection or vaccination.

## Induction, Persistence, and Function of CD4 T_RM_ Cells in Infection

**Figure 1:**
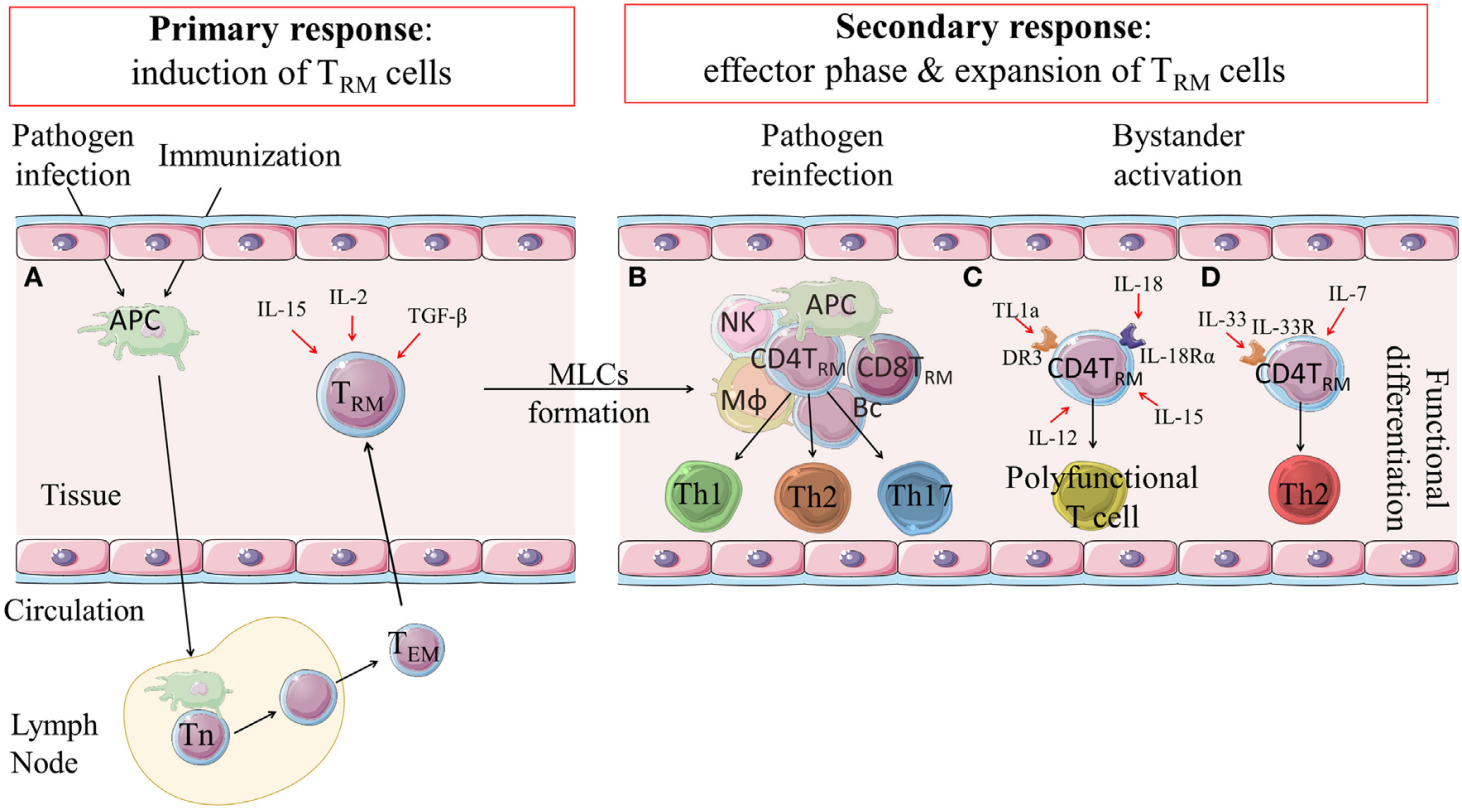
Induction, expansion and function of CD4 T_RM_ cells. **(A)** Following infection or immunization effector and memory T cell responses are induced in lymph nodes. Effector-memory T cells can migrate to the tissue where they are retained as CD4 T_RM_ cells through expression of integrins and adhesion molecules, such as CD44, CD69 and/or CD103. Their induction is influenced by IL-2, IL-15 and TGF-β. **(B)** CD4 T_RM_ cells are retained in the tissues and can be reactivated locally in MLCs during secondary responses following reinfection or after infection in an immunized host. CD4 T_RM_ cells are expanded and can become effector Th1, Th17 or Th2-type cells that mediate rapid clearance of the infection. **(C)** There is emerging evidence that CD4 T_RM_ cells can be non-specifically activated in the tissues by cytokines, such as IL-18, IL-12, IL-15 and TL1a, and develop into polyfunctional T cells that have potent anti-pathogen activity. **(D)** It has also been suggested that IL-7 and IL-33 may non-specifically activate CD4 T_RM_ cells to become effector Th2 cells. DR3: death receptor 3; TL1a: tumor necrosis factor (TNF)-like cytokine 1a; MLCs: memory lymphocyte clusters; APC: antigen presenting cell; Mϕ: macrophage; NK: natural killer cell; Bc: B cells; Th: T helper (cell); T_n_: naïve T cell; T_EM_: effector memory T cells; T_RM_: tissue-resident memory T cells.

The key function of T_RM_ cells is to rapidly respond to infection or reinfection with a pathogen and to orchestrate local immune responses in the tissue that mediate clearance of the pathogen. T_RM_ cells that are generated by infection are sustained in the local tissue after clearance of the pathogen (15). During a life time, T_RM_ cells accumulate in many tissues and provide long-term local protection against subsequent infection by reactivation with specific antigen (15, 16). The persistence of T_RM_ cells in tissue after pathogen clearance and the mechanism of maintenance in the tissues is unclear. Although memory T cells classically require antigen-specific stimulation through the T cell receptor (TCR) and costimulation for proliferation, there is evidence that T_RM_ cells may respond in an innate-like manner to cytokines, including IL-18, IL-12, IL-15, and tumor necrosis factor (TNF)-like cytokine 1A (TL1a), without TCR activation (17). Signaling from IL-15 and TGF-β has been shown to be critical for persistence of mature CD8 T_RM_ cells (Figure 1) (18). Furthermore, while much of the focus has been on pathogen-induced T_RM_ cells, these cells can also be generated by non-infectious agents, including allergens or autoantigens, and can mediate pathology in asthma or autoimmune disorders (19, 20). Nonetheless, there is growing evidence from mouse studies of a beneficial role for CD4 and CD8 T_RM_ cells in protection against a variety of infectious pathogens.

### Viral Infections

CD8 cytotoxic T lymphocytes play a critical role in the control of viral infections by killing virally infected cells. However, CD4 T cells also play a vital role in protective immunity to viruses by helping antibody production and facilitate the induction and expansion of virus-specific memory CD8 T cells, including CD8 T_RM_ cells (21–23). Recent studies demonstrated that respiratory infection with a number of different viruses can also induce CD4 T_RM_ cells. It has been reported that influenza virus infection induces polyclonal, virus-specific memory CD4 T cells in the lungs and the spleens of infected mice (24). Adoptive transfer experiments with CD4 T cells specific for influenza hemagglutinin (HA) showed that lung-derived memory CD4 T cells were almost exclusively found in the lungs 7 days after transfer to naïve mice. In contrast, transferred splenic memory CD4 T cells were distributed in multiple tissues and were not retained in these tissues. Furthermore, only HA-specific CD4 T_RM_ cells from the lungs conferred protection against lethal influenza infection (24). These findings suggested that lung-resident CD4 T_RM_ cells, but not spleen memory CD4 T cells, play a crucial role in local protective immunity against influenza virus infection in the lungs.

Antigen-specific T_RM_ cells induced during influenza virus infection are localized near the airways and bronchovascular bundles and are maintained long after viral clearance independently of replenishment from lymphoid stores (25). IL-2-dependent and -independent mechanisms have been described for generation of influenza-specific CD4 T_RM_ cells, contributing to heterogeneity of protective T_RM_ cells. The formation of IL-2-independent subset of T_RM_ cells required a direct IL-15 signal to CD4 T-cell effectors (26, 27). Similarly, intranasal infection of mice with lymphocytic choriomeningitis virus (LCMV) required IL-2 signaling for the generation of virus-specific CD4 T_RM_ cells. CD4 T cells that lacked CD25, the IL-2 receptor α chain, failed to develop into lung T_RM_ cells in LCMV-infected mice (28). These studies suggest a broad mechanism involving IL-2 signaling pathway for the formation of CD4 T_RM_ cells.

Similar to respiratory tissue, the female reproductive track is vulnerable for repeated infections. In a model of herpes simplex virus-2 (HSV-2) infection, where thymidine-kinase defective (TK^−^) HSV-2 was used to avoid neurovirulence (29), CD4 T cells infiltrated the female genital mucosa during infection and provided help for mobilizing cytotoxic effector CD8 T cells that cleared the infection (30). In addition, infection with TK^−^ HSV-2 provided local, long-term protection against a secondary infection with wild-type HSV-2 based on the formation of CD4 T_RM_ cells, which were retained mainly among memory lymphocyte clusters (MLCs) (31). Therefore, an effective vaccine against HSV-2 infection may be possible by targeting the induction of T_RM_ cells.

### Bacterial Infections

A number of recent studies have indicated that CD4 T_RM_ cells established in non-lymphoid tissues after primary infection provide protection against reinfection with the same pathogen. In a mouse model of *B. pertussis* infection, it was demonstrated that transfer of Th1-like cells resulted in pathogen clearance in the absence of specific antibodies (32). We have recently reported that infection of mice with *B. pertussis* induce the development of CD69^+^CD103^+/−^ CD4 T_RM_ cells in the lungs (7). Treatment of convalescent mice with FTY720 did not affect clearance of a secondary infection with *B. pertussis*, suggesting that an established population of T_RM_ cells mediates local protective immunity against reinfection. Moreover, adoptive transfer of CD4 T_RM_ cells from the lungs of convalescent mice conferred protection against *B. pertussis* infection in naïve mice (7). It has also been demonstrated that pulmonary infection with *Mycobacterium tuberculosis* is controlled by a subset of lung parenchymal-homing CD4 T cells. Adoptive transfer of parenchymal T_RM_ cells into susceptible T cell-deficient hosts showed preferential migration back to the lung and superior control of infection compared with the intravascular CD4 T cells (33).

In a mouse model of pneumonia, repeated respiratory infections with *Streptococcus pneumoniae* (pneumococcus) seeded the lungs with antibacterial CD4 T_RM_ cells that mediated heterotypic protection (34). Furthermore, oral infection of mice with *Listeria monocytogenes* induced robust pathogen-specific CD4 T cell response, the majority of which migrated to the intestine and were transitioned to long-lived T_RM_ cells with a polyfunctional Th1 profile, secreting predominantly IFN-γ, TNF, and IL-2, and detectable level of IL-17 (35).

There is also emerging data to suggest that CD4 T_RM_ cells play a central role in protection against *Chlamydia trachomatis* infection (36). It has been shown that lymphoid aggregates, which contained CD4 T cells, are formed in the genital tract of mice during infection with *C. trachomatis*. These aggregates, which resembled MLCs described by Iijima and Iwasaki (31), persisted long after the infection had resolved (37). The formation of lymphoid aggregates with T_RM_ cells during primary infection provided a robust response to secondary infectious challenge and was dependent on B cell antigen presentation in established MLCs (38). These findings demonstrate that bacterial infection at various mucosal site (lungs, gut, and gential tract) induce CD4 T_RM_ cells that mediate protective immunity against reinfection of the mucosa with the relevant pathogen.

### Parasite Infection

The development of Th2-type immune responses are required for protective immunity against infection with helminths, such as *Nippostrongylus brasiliensis* (39). Recent studies on lung infection with *N. brasiliensis* revealed that a Th2-type polarized pulmonary CD4 T cell population established during infection and can drive effective local adaptive immunity to reinfection with the same parasite (40). In a mouse model of intestinal infection with *Heligmosomoides polygyrus*, functional memory Th2 cells persisted in the lamina propria and the peritoneal cavity after resolution of infection. Interestingly, cells at both locations produced Th2 cytokines after restimulation; however, only peritoneal CD4 T_RM_ cells mediated protective immunity against the helminth infection. The Th2-type CD4 T_RM_ cells expressed high levels of the IL-33 receptor and produced effector cytokines in response to IL-33 and IL-7 independently to TCR activation (41). CD4 T_RM_ cells have also been identified in the skin after infection with *Leishmania major* where they persisted long after the pathogen was cleared (42, 43). Interestingly, CD4 T_RM_ cells were also found in the flank skin far from the primary infection site in the ear. Pathogen-specific CD4 T_RM_ cells produced IFN-γ in response to secondary infection and rapidly recruited other memory cells from the circulation; however, recruitment and activation of inflammatory monocytes was required for optimal protection (42, 43). These findings suggest that Th1- and Th2-type T_RM_ cells are induced by infection with different parasites and these cells mediate host protective immunity against the relevant parasite.

### Distinct Subtypes of Infection-Induced T_RM_ Cells

A key research question that is beginning to be addressed is whether there are distinct Th1, Th2, and Th17 subtypes of T_RM_ cells and whether effector Th1, Th2, and Th17 arise from T_RM_ cells in the tissues after reinfection with a pathogen. It has been reported that skin infection with *C. albicans* in humans or mice leads to formation of IL-17-producing CD4 T_RM_ cells that reside in papillary dermis and rapidly clear the infection after re-exposure to the pathogen (6). It was also shown that protection against oropharyngeal candidiasis is mediated by oral-resident natural Th17 cells (44). Th1 cells have an established protective role in immunity to viruses and intracelluar bacteria and evidence is emerging that IFN-γ-seceting T_RM_ cells are critical for long-term protection against these pathogens. The findings from the parasite field also suggest that Th2 or Th1-type T_RM_ cells may play key roles in protective immunity against extracellular and intracellular parasites, respectively. However, the factors that control the development or specific activation of effector Th1, Th2, and Th17 from T_RM_ cells in the tissues after reinfection with a pathogen are still unclear (Figure 1).

## Vaccine-Induced T_RM_ CD4 T Cells

While most successful vaccines in use today mediate protective immunity through the induction of antibodies, optimum protection against many pathogens requires the generation of appropriate cellular immune responses, including CD4 T cells. Indeed, there are increasing number of studies showing that the formation of CD4 T_RM_ cells after natural infection mediates protective immunity against secondary exposure to the same pathogen. Although there is less evidence of a role for CD4 T_RM_ cells in protective immunity generated with vaccines in use today, the recent studies in mice have suggested that the induction of CD4 T_RM_ cells may be central to persistent vaccine-induced protection against a range of mucosal pathogens. Immunization approaches that induce systemic and tissue-retained memory CD4 T cells may be critical to persistent protection, because they are long-lived in the tissues and are more polyclonal than CD8 T cells (45, 46). It has also been suggested that CD4 T cell are less prone than CD8 T cell to immune escape from antigenic variation in T cell epitopes (47). Therefore, the induction of CD4 T_RM_ cells may be a promising approach for the design of new or improved vaccines. In the light of recent findings on the development of T_RM_ cells in different mucosal tissues, several important factors have to be considered in the development optimal immunization approaches for the induction of these cells.

## Location, Compartmentalization, and Route of Immunization

The efficacy of certain vaccines is influenced by the route of immunization. A comparison of two different licensed influenza vaccines given by intranasal or parenteral routes demonstrated that the route of administration, as well the type of vaccine (live versus killed), influenced the induction of CD4 T_RM_ cells. Intranasal administration of attenuated influenza virus vaccine (FluMist) generated CD4 T_RM_ cells in the lungs, which mediated long-term protection against non-vaccine strains of influenza virus. In contrast, an inactivated influenza virus vaccine (Fluzone) induced strain-specific neutralizing antibodies, but failed to induce T_RM_ cells, even when delivered intranasally (48). Studies with coronaviruses (CoVs), which cause a severe respiratory disease in humans, showed that intranasal, but not subcutaneous, immunization with SARS-CoV nucleocapsid (N) protein induced airway and lung-parenchymal antigen-specific memory CD4 T_RM_ cells (49). However, protection was lost following depletion of airway, but not parenchymal, memory T_RM_ cells. These results provide evidence of compartmentalization of the immune response induced by vaccination and suggest that T_RM_ cells may preferentially populate the site of induction/immunization.

Vaccine-induced T_RM_ cells can be localized not only near the site of immunization but can be spread to other parts of the same tissue. Mucosal vaccination can induce broad mucosal-tropic memory lymphocytes. Intranasal immunization with attenuated TK^−^ HSV-2 resulted in long-lasting protection mediated by HSV-2-specific CD4 T_RM_ in distant tissues, the vaginal mucosa (50, 51). Similarly, transmucosal protection against *Chlamydia muridarum* infection was established after oral vaccination. Colonization of the gastrointestinal tract with non-pathogenic bacterium induced protective immunity in the genital tract (52). Furthermore, it was shown that intranasal, but not subcutaneous, vaccination with ultraviolet light (UV)-inactivated *C. trachomatis* complexed with charge-switching synthetic adjuvant particles induced protective CD4 T cells that rapidly populated uterine mucosa with T_RM_ cells (53).

Conserved vaccine antigens have the potential to induce broadly cross-protective immunity against many strains of the same pathogen. This is particularly important for pathogens like influenza virus, where the HA molecule, the target antigen for neutralizing antibodies, undergoes significant antigen variation allowing escape from protective immunity against seasonal strains of influenza virus. It was reported that intranasal immunization with influenza virus matrix protein ectodomain (M2e) adjuvanted with CTA1-DD generated highly protective M2e-specific lung-resident Th17 T_RM_ cells (54). Moreover, immunized mice were protected against a potentially lethal challenge with H3N2 or H1N1 influenza virus strains, demonstrating effective cross-protection. These results demonstrate that induction of T_RM_ cells and their ability to protect against mucosal infections is influenced by the route of immunization. Therefore, the design of more effective vaccines against mucosal pathogens needs to move beyond the common approach of using injectable vaccines and should utilize appropriate routes of mucosal immunization to promote protective T_RM_ cells at the sites of infection.

## Role of Adjuvants and Antigens in Vaccine-Induced T_RM_ Cells

The choice of adjuvant can influence the induction of cellular immune response and formation of T_RM_ cells following vaccination. Stary et al. showed that genital infection with *C. trachomatis* induced protective immunity in the uterus, whereas immunization with UV-inactivated *C. trachomatis*, which favored generation of regulatory T cells, exacerbated subsequent infection (53). However, an experimental vaccine comprising UV-inactivated *C. trachomatis* complexed with charge-switching synthetic adjuvant particles was effective at inducing antigen-specific CD4 T_RM_ cells and long-term protection (53). It has been reported that IL-1β may act as an adjuvant for the induction of T_RM_ that mediate protective immune responses against influenza virus infection. Intranasal administration of a novel vaccine, based on recombinant adenoviral vectors (rAd) encoding influenza HA and nucleoprotein in combination with rAd-IL-1β promoted the generation of CD103^+^CD69^+^ T_RM_ cells that mediated protection against infection with homologous and heterologous influenza virus strains (55).

Current vaccines against whooping cough (pertussis) are administered parenterally, usually by intramuscular route; however, immunity is relatively short lived, especially after immunization with acellular pertussis (aP) vaccines, which is administered with alum as the adjuvant (56). Studies in a baboon model have shown that the current aP vaccine fails to prevent nasal colonization and transmission of *B. pertussis* (57). In contrast, immunization with an attenuated *B. pertussis* vaccine, BPZE1, protected baboons against nasopharyngeal colonization and disease induced by a highly virulent strain of *B. pertussis* (58). Since BPZE1 is replicating bacterium delivered by the intranasal route, it is likely to induce respiratory T_RM_ cells. Current parenterally delivered aP vaccines preferentially induce strong antibody and Th2-type responses, whereas experimental aP vaccines formulated with more potent adjuvants, such as TLR agonists, induce potent Th1 and Th17 responses in mice (59, 60). Therefore, it should also be possible to develop an intranasally delivered aP vaccine with an appropriate adjuvant that induces IL-17 and IFN-γ-secreting T_RM_ cells in the lungs and nasal tissue. It has been reported that the formation of CD8 T_RM_ cells in the nasal epithelium after immunization are key for limiting influenza viral spread to the lower respiratory track (61). Therefore, induction of respiratory T_RM_ cells by intranasal immunization appears to be an ideal approach for inducing long-term protection in the upper and lower respiratory tract.

## Implications for New or Improving Vaccine Design

In the vaccine field, the big questions include (1) whether CD4 T_RM_ cells are really important for long-term protective immunity in humans, (2) how antigen-specific T_RM_ cells can be optimally induced by vaccination, and (3) how antigen-specific T_RM_ cells can be detected and quantified after infection or vaccination in humans. Most of the vaccines in use today protect by induction of antibody responses that either neutralize viruses or bacterial toxins or opsonize bacteria for killing by phagocytic cells. However, there are other infectious diseases, such as HIV, malaria, and tuberculosis where we do not have an effective vaccine, and where T-cell responses may be more important in preventing or clearing the infection. Furthermore, there is a move away from killed and live attenuated vaccine to subunit vaccines, which are usually delivered by injectable routes. However, the first choice adjuvant alum, while capable of promoting the induction of antibody and Th2 responses is not very effective at inducing Th1 responses. In addition, injected alum-adjuvant vaccines do not appear to be capable of inducing T_RM_ cells. The current pertussis aP vaccine is a good example; it fails to induce Th1 cells (59) and protective immunity wanes rapidly after immunization in children (62). This is likely to reflect a failure to induce CD4 T_RM_ cells. Effort to develop more effective aP and other subunit vaccines need to focus on mucosal routes of immunization and adjuvants that induce T_RM_ cells, as well Th1 and Th17 cells that can be detected in the periphery. It was reported that subcutaneous priming followed by intranasal boosting with group A streptococcal C5a peptidase formulated with a cationic adjuvant induced persistent local immune response including IgA, Th17 cells, and T_RM_ cells (63). The “Prime and Pull” strategy may be a useful approach for eliciting both systemic and local immunity and immunological memory with subunit vaccines (64).

The vast majority of the published work on T_RM_ cells have been based on studies in mouse models. CD69^+^ T_RM_ cells have also been identified in human tissues (65). However, since T_RM_ cells are in the tissue rather than the blood, one of the challenges in translating the mouse studies to humans is the difficulty in getting routine access to human mucosal tissue samples to study and quantify the induction of T_RM_ cells following infection or vaccination. This could be overcome by the identification of precursors of T_RM_ in the circulation as they migrate from lymph nodes to tissues. Peripheral memory CD8 T cells that express CX3CR1 have been identified in mice (66). However, it has also been reported that intravascular CX3CR1^+^KLRG1^+^ Th1 cells did not migrate into the lungs and were unable to control *M. tuberculosis* infection (67). Nevertheless, the design of new or improved vaccines that confer sustained sterilizing immunity at mucosal surface will be greatly facilitate by the identification of immunization approaches that induce potent pathogen-specific T_RM_ at the mucosal site of infection.

## Author Contributions

KM and MW co-authored this article.

## Conflict of Interest Statement

The authors declare that the research was conducted in the absence of any commercial or financial relationships that could be construed as a potential conflict of interest.
